# Neonatal Liver Failure and Congenital Cirrhosis due to Gestational Alloimmune Liver Disease: A Case Report and Literature Review

**DOI:** 10.1155/2017/7432859

**Published:** 2017-01-30

**Authors:** Carolina Roos Mariano da Rocha, Renata Rostirola Guedes, Carlos Oscar Kieling, Marina Rossato Adami, Carlos Thadeu Schmidt Cerski, Sandra Maria Gonçalves Vieira

**Affiliations:** ^1^Medical School, Universidade Federal do Rio Grande do Sul, Porto Alegre, RS, Brazil; ^2^Hospital de Clínicas de Porto Alegre, Pediatric Gastroenterology Unit, Porto Alegre, RS, Brazil; ^3^Department of Pathology, Hospital de Clínicas de Porto Alegre, Pathology Unit, Universidade Federal do Rio Grande do Sul, Porto Alegre, RS, Brazil; ^4^Department of Pediatrics, Hospital de Clínicas de Porto Alegre, Pediatric Gastroenterology Unit, Universidade Federal do Rio Grande do Sul, Porto Alegre, RS, Brazil

## Abstract

Neonatal liver failure (NLF) is a major cause of neonatal morbidity and mortality, presenting as acute liver failure and/or congenital cirrhosis. Many affected patients show antenatal signs of fetal injury. There are several causes of NLF and early diagnosis is mandatory to elucidate the etiology and determine a specific treatment or the best management strategy. Gestational alloimmune liver disease associated with neonatal hemochromatosis (GALD-NH) is a rare but potentially treatable cause of NLF. It should be considered in any neonate with fetal signs of disease and postnatal signs of liver failure with no other identifiable causes. GALD-NH is often diagnosed late and patients are therefore referred late to specialized centers, delaying treatment. This case highlights the consequences of late diagnosis and treatment of GALD-NH and emphasizes the importance of a high grade of suspicion of this disease in order to refer the patient to a specialized center soon enough to perform the appropriate treatment.

## 1. Introduction

Liver disease in early infancy encompasses a wide spectrum of conditions, including infectious, metabolic, and hematologic disorders, congenital vascular and heart diseases, drug-related toxicity, hypoxia, and gestational alloimmune liver disease associated with neonatal hemochromatosis (GALD-NH) [1]. Congenital cirrhosis is a rare disorder of the liver and is defined as cirrhosis identified at or shortly after birth [2].

GALD-NH, previously known as neonatal hemochromatosis, typically presents as subacute fetal liver injury and/or congenital cirrhosis [3]. Liver injury begins during intrauterine life and most patients with GALD-NH show signs of fetal disease. Infants are often hypoglycemic and present with coagulopathy, hypoalbuminemia, jaundice, and edema within the first few hours to days after birth [3]. GALD-NH was once considered a lethal disease. However, neonatal treatment with exchange transfusion and intravenous immunoglobulin (IVIG) has now reduced the need for liver transplantation (LTx) and improved the prognosis. Despite this strategy, GALD-NH is still associated with high perinatal morbidity and mortality rates [3]. The diagnosis of GALD-NH remains challenging and requires a high grade of suspicion.

We report a case of neonatal liver failure (NLF) in a preterm, growth-restricted infant who underwent extensive investigation and was diagnosed postmortem as having congenital cirrhosis due to GALD-NH. Issues related to liver failure in early infancy are then discussed.

## 2. Case Report

We describe a case of a female infant born healthy by cesarean section at 31 + 4 weeks of gestation with a birth weight of 835 g. The 1- and 5-minute Apgar scores were 6 and 8, respectively. Antenatal examination revealed intrauterine growth restriction and severe oligohydramnios. Parents were nonconsanguineous and mother had no previous pregnancies and denied any family history of neonatal death or chronic diseases.

Shortly after birth, she required mechanical ventilation and presented anemia, coagulopathy, and unconjugated hyperbilirubinemia. During the following days, she developed recurrent hypoglycemia, cholestatic jaundice, ascites, hepatomegaly, and lower gastrointestinal bleeding.

On admission to our unit, she was 85 days old and weighed 2040 g. Clinical examination revealed a jaundiced infant with edematous lower limbs and a systolic murmur. Abdominal collateral circulation, grade 2 ascites, and hepatosplenomegaly were present. Laboratory tests showed anemia, thrombocytopenia, abnormal synthetic liver function (hypoalbuminemia and coagulopathy), and a mild increase in liver enzymes. The infant had elevated serum ferritin and abnormal iron profile (Table 1). Abdominal ultrasound showed gaseous distention of the bowel, moderate-volume ascites, and multiple hepatic nodules. Ascitic fluid analysis ruled out spontaneous bacterial peritonitis and suggested portal hypertension (serum-ascites albumin gradient [SAAG] ≥  1.1) (Table 2). Transthoracic echocardiography showed stenosis of small pulmonary vessels and interventricular septal defect with hemodynamic repercussion. Causes of liver disease, including IEM, were investigated but yielded no definite diagnosis (Table 2).

**Figure 1:**
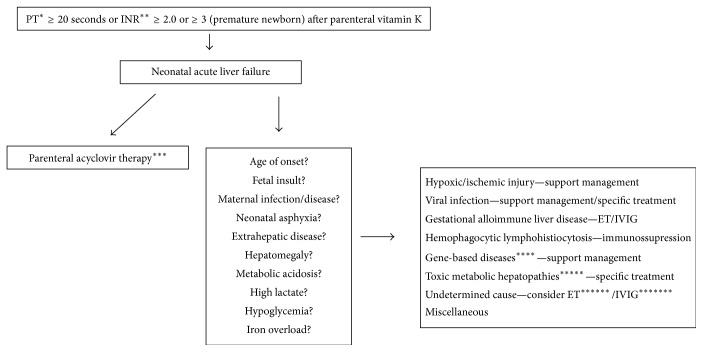
Simplified diagnostic and management algorithm for acute liver failure in neonates. ^*∗*^PT is prothrombin time; ^*∗∗*^INR is international normalized ratio; ^*∗∗∗*^herpes simplex virus is the most common virus associated with NALF with high mortality rate without early initiation of treatment (cutaneous findings are uncommon); *∗∗∗∗* includes respiratory chain defects, errors in fatty acid oxidation, and mitochondrial DNA depletion syndromes; *∗∗∗∗∗* includes hereditary tyrosinemia type 1, galactosemia, and hereditary fructose intolerance; ^*∗∗∗∗∗∗*^ET is exchange transfusion; ^*∗∗∗∗∗∗∗*^IVIG is intravenous immunoglobulin (modified from Taylor and Whitington, 2016).

**Figure 2:**
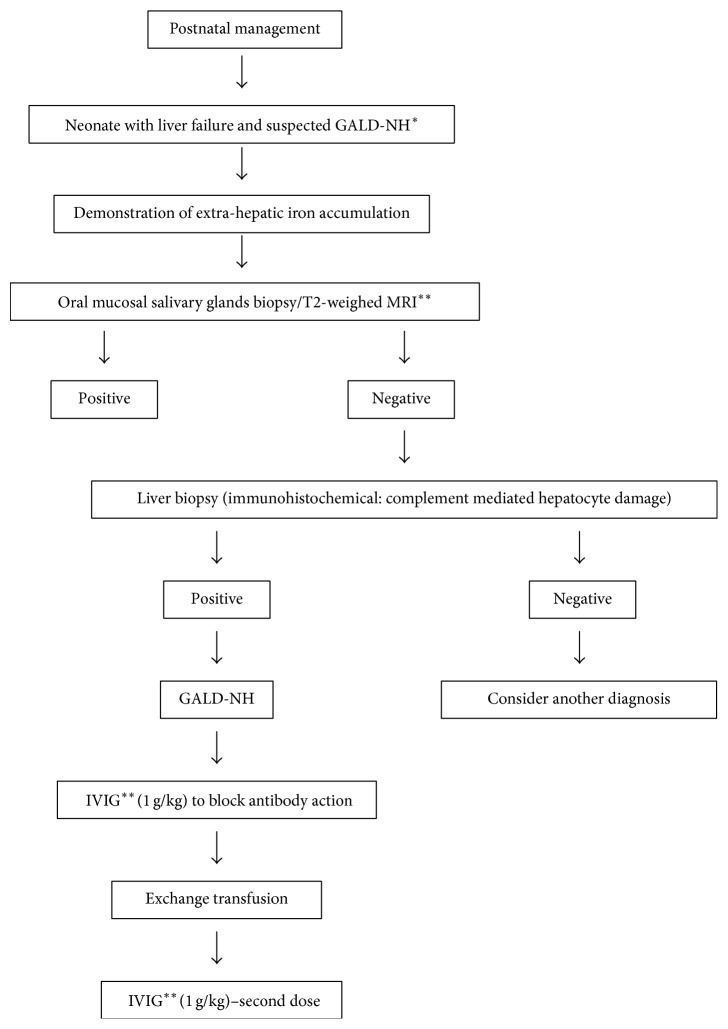
Diagnostic and management algorithm for gestational alloimmune liver disease-neonatal hemochromatosis. ^*∗*^GALD-NH is gestational alloimmune liver disease-neonatal hemochromatosis; ^*∗∗*^IVIG is intravenous immunoglobulin (modified from Lopriore et al., 2013).

Based on clinical, biochemical, and serological findings and genetic screening tests, the diagnosis of GALD-NH was considered. Magnetic resonance imaging (MRI) and mucosal salivary gland biopsy were performed to detect extrahepatic iron deposition. Both were negative for extrahepatic siderosis. Needle biopsy of the liver revealed hepatocanalicular cholestasis and sinusoidal fibrosis. Figures 1 and 2 show a diagnostic algorithm for NLF and GALD-NH, respectively.

There was progressive hepatic dysfunction, with severe coagulopathy, hypoalbuminemia, anasarca, and renal impairment. The patient was evaluated for LTx but ultimately did not undergo the procedure due to the development of sepsis. There was progressive multiple organ failure and, 90 days after admission, the patient died.

**Figure 3:**
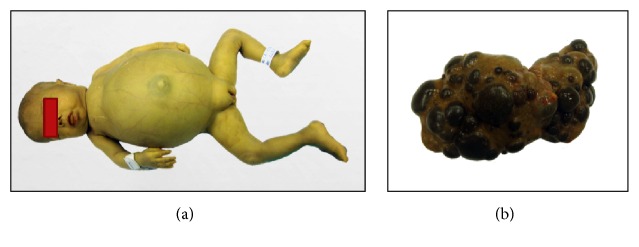
(a) Overview of the patient. (b) Macroscopic view of the liver at autopsy. Analysis of the liver showed multiple nodules in the parenchyma, causing architectural distortion.

**Figure 4:**
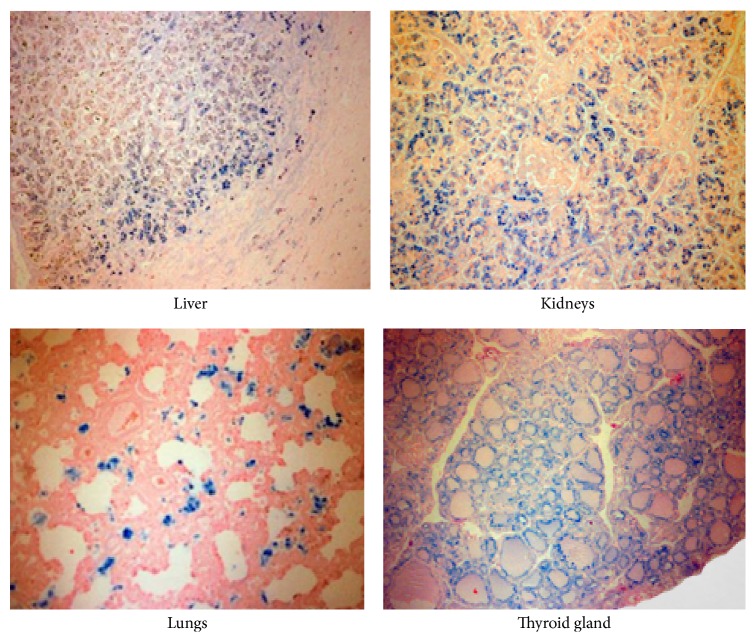
Perls' stain showing hepatic and extrahepatic siderosis.

Autopsy was performed with formal permission from parents and the diagnosis of GALD-NH was confirmed by the presence of multisystem hemosiderin deposition (liver, pancreas, lungs, gastrointestinal tract, heart, choroid plexus, adrenal glands, and thyroid gland), massive loss of liver parenchyma, marked cholestasis, and severe hemosiderin deposition with significant ductular proliferation (ductular reaction). The liver weighed 136 g, with multiple brownish-green nodules distorting its architecture (Figures 3 and 4).

## 3. Discussion

We reported a case of NLF in an infant with antenatal signs of disease, such as prematurity, oligohydramnios, and intrauterine growth restriction. Shortly after birth, the infant developed hepatosplenomegaly, cholestasis, and grade 2-3 ascites, suggesting chronic liver injury.

NLF, represented by failure of synthetic liver function within 4 weeks of birth, is rare. The diagnosis may be difficult to make and the mortality rate is invariably high [1, 4]. The diagnosis of NLF can be defined based on the following criteria: (1) hepatic-based coagulopathy (prothrombin time [PT] ≥ 15 s or international normalized ratio [INR] ≥ 1.5 not corrected by vitamin K in the presence of clinical hepatic encephalopathy [HE] or PT ≥ 20 s or INR ≥ 2 regardless of clinical HE); (2) biochemical evidence of acute liver injury; and (3) no known evidence of chronic liver disease [4]. It is important to highlight that HE is difficult to diagnose in children, and thus it is often diagnosed late, mainly because of the subjectivity of the diagnostic criteria [5]. Although our patient met the criteria (1) and (2) described above, she showed clinical signs of chronic liver injury, suggesting congenital cirrhosis secondary to a prenatal insult. Therefore, our patient was an infant with failure of synthetic liver function in a scenario of chronic liver disease. Identifying the etiology of NLF is important in order to define management strategies, prognosis, and the risk of recurrence in subsequent pregnancies [6, 7]. Based on our experience and literature reports, we suggest algorithms for diagnosis and management of NLF and GALD-NH (Figures 1 and 2).

Among the various causes of NLF, some are worthy of consideration and further discussion. Antenatal/perinatal infection with herpes viruses, adenovirus, parvovirus B19, and hepatitis B virus are potential causes of NLF. Herpes simplex virus is the most common viral etiology of NLF, carries a high mortality rate, and is rarely accompanied by skin lesions. Transplacental infection usually results in generalized fetal infection, but it is also often associated with multisystem involvement, including the central nervous system [8]. IEM do not affect the fetus, but usually manifest in the postnatal period. IEM must be promptly investigated because dietary management or specific treatment can be lifesaving. Tyrosinemia, hereditary fructose intolerance, and galactosemia are the most common metabolic diseases to be considered. Tyrosinemia is caused by a defect in the final enzyme of the tyrosine degradation pathway. Liver failure, ascites and edema with onset in the first weeks or months of life are common. Unlike the present case, jaundice is usually mild [9]. Classic galactosemia is an autosomal recessive disorder of carbohydrate metabolism caused by a severe deficiency of the enzyme galactose-1-phosphate uridyltransferase (GALT). Upon consumption of lactose, the affected infants develop a severe condition with multiorgan involvement, including liver failure [10]. GALD-NH usually manifests as antenatal liver disease and is discussed in more detail below. In the present case, although antenatal presentation and absence of significant extrahepatic involvement were considered, several etiologies of NLF were ruled out by comprehensive laboratory investigations, imaging studies, and histopathological analysis.

GALD-NH, although rare, is the main cause of NLF and/or congenital cirrhosis in neonates [8]. The exact incidence of the disease remains unknown. Liver injury begins during intrauterine life and is caused by the active transport of anti-fetal liver IgG antibodies from mother to fetus starting around 12 weeks of gestation, which activates the terminal complement cascade and results in hepatocyte injury and death [7, 8, 11]. The alloimmune hypothesis has been confirmed by studies showing the involvement of complement in the pathogenesis of hepatocyte injury, which could result only from maternal alloimmunity [8].

Antenatal manifestations include growth restriction, prematurity, hydrops fetalis, oligohydramnios, fetal hepatomegaly, and ascites [3, 8]. There is often a maternal sibling history of loss of stillbirth or neonatal disease [3]. As observed in the present case, the initial presentation may mimic that of any of the many causes of NLF, making the diagnosis challenging to the medical team [8]. Postnatal clinical manifestation is typically subacute liver injury and/or congenital cirrhosis, characterized by marked coagulopathy and recurrent hypoglycemia within a few hours or days after birth, followed by progressive edema, hypoalbuminemia, ascites, jaundice, and renal impairment [7, 8]. Laboratory investigation shows typical signs of liver failure: coagulopathy, hypoalbuminemia, hyperammonemia, and hypoglycemia. Aminotransferase levels are relatively low, rarely exceeding 100 IU/L. The levels of conjugated and unconjugated bilirubin, serum ferritin, serum iron, and transferrin saturation are elevated. Elevated alpha-fetoprotein is also present.

In the case reported here, GALD-NH was recognized as an important diagnosis to be considered. Therefore, an attempt was made to demonstrate extrahepatic siderosis by mucosal salivary gland biopsy and T2-weighted MRI. Both were negative. While a positive MRI or biopsy finding of extrahepatic siderosis confirms the diagnosis of GALD-NH, a negative finding does not rule it out. Hepatic siderosis can be present in many other causes of fulminant liver failure in neonates. However, siderosis of extrahepatic tissues is not found in neonatal diseases other than GALD-NH, and inhibition of membrane attack complex formation (complement C5b-9 complex) on hepatocytes is taken as evidence of alloimmune injury; however, this test remains a research tool and is not yet available for clinical use [3, 8]. Liver biopsy was performed to exclude other diagnoses and assess liver fibrosis [12]. Postmortem examination is an integral part of the diagnostic evaluation and should be performed in any infant with conditions suggestive of GALD-NH [6]. Although our patient showed many of the antenatal and neonatal manifestations of GALD-NH, unfortunately, the diagnosis was made only after autopsy.

When GALD-NH is suspected, the patient should be promptly referred to a specialized pediatric liver center. In the past, treatment was based on the use of antioxidants and chelation therapy, which failed to produce successful results over the years [8, 13]. Nowadays, the standard treatment for GALD-NH is directed toward the alloimmune etiology, including IVIG and exchange transfusion [3, 7]. Exchange transfusion is used to remove existing reactive antibody and IVIG (1 g/kg) is administered to block antibody action and interfere with complement activation.

The fact that our patient received no specific treatment deserves some comment for learning. On admission, our patient was 85 days old and had evidence of severe hepatic decompensation. She was classified as Child-Pugh C (score of 10) with a PELD score of 29, rapidly progressing to refractory ascites. Because of the severity of liver disease, the advanced age of the patient, and the absence of extrahepatic siderosis, we directed our efforts to indicating LTx and, therefore, medical treatment was not performed immediately. The patient was considered to be “too sick to treat” at that time and, unfortunately, died while waiting for a transplant. We cannot state whether IVIG and exchange transfusion would have changed the prognosis. According to Whitington, the plasticity of the neonatal liver affected with GALD-NH allows recovery even from severe injury, but the efficacy of medical treatment in reversing liver disease remains uncertain [13]. After this case, in our unit, IVIG is given to all infants with NLF and suspected GALD-NH despite the absence of extrahepatic siderosis [11].

The rate of lethal recurrence in subsequent pregnancies of a woman who has had an infant affected with GALD-NH is 90% without intervention. This rate can be changed by antenatal treatment with IVIG, given at 14 weeks, at 16 weeks, and weekly from 18 weeks of gestation until delivery [7, 8].

In view of the generally poor prognosis, GALD-NH is a frequent indication for LTx in neonates [7]. However, evidence shows that the current standard treatment for GALD-NH is resulting in a significant reduction in the need for LTx, which can be demonstrated by the survival rate of 75% without LTx when the combination of exchange transfusion and IVIG is used as treatment [12].

## 4. Conclusion

GALD-NH is a rare but important cause of severe NLF and should be suspected if there is evidence of fetal injury with no other definable cause. A high grade of suspicion is the key for the diagnosis of GALD-NH, and early use of IVIG should not be postponed in order to allow longer survival of the native liver.

## Figures and Tables

**Table 1:** Serum and ascites laboratory values on admission.

*Serum laboratory analysis*	
Hemoglobin/hematocrit	7.2 g/dL (10–13)/20.1% (33–41%)
MCV	77.5 fL (80–98)
WBC count	7,470 (5,000–14,000)/1% myelocytes; 3% blasts; 79% neutrophils (20–40%); 13% lymphocytes (50–80%)
Platelet count	67,000 (200,000–550,000)
ALT/AST	71 U/L (<41)/172 U/L (<40)
TB/CB	11.9 mg/dL (0.3–1.2)/9 mg/dL (<0.2)
INR	2.01
Albumin	2.9 g/dL (3.2–4.9)
Factor V	49.6 (70–120)
GGT	178 U/L (<38)
Alkaline phosphatase	878 U/L (82–383)
Creatinine	0.3 mg/dL (0.6–1.1)
Serum ferritin	>1,650 (10–291)
Serum iron	126 ug/mL (50–170)
Transferrin saturation	70% (25–50)
Fibrinogen	222 mg/dL (200–400)
Ammonia	96 umol/L (11–32)
*Ascitic fluid analysis*	
Cellularity	65 cells: 39 RBCs; 2% neutrophils; 69% macrophages; 28% lymphocytes
Albumin	0.6 g/dL → SAAG = 2.2
Total protein	1 g/dL
Triglycerides	17 md/dL
Amylase	9 U/L
Culture	Negative

MCV = mean corpuscular volume; WBC = white blood cell; ALT = alanine aminotransferase; AST = aspartate aminotransferase; TB = total bilirubin; CB = conjugated bilirubin; INR = international normalized ratio; GGT = gamma-glutamyl transpeptidase; RBCs = red blood cells; SAAG = serum-ascites albumin gradient.

**Table 2:** Causes of neonatal liver failure excluded and assessment methods.

Cause	Assessment method
Syphilis	Quantitative nontreponemal serologic test
Toxoplasmosis	Serology (IgG and IgM)
Rubella	Serology (IgG and IgM)
Cytomegalovirus	Serology (IgG and IgM) and urinary PCR
Herpes simplex virus	Serology (IgG and IgM)
Parvovirus B19	PCR
Infection/sepsis	Blood culture, urine culture, and chest X-ray
Hepatitis viruses A, B, and C	Serology (IgG and IgM)
Tyrosinemia	Urinary succinylacetone
Galactosemia	Galactose-1-phosphate uridyltransferase
Hereditary fructose intolerance	Transferrin isoelectric focusing
Niemann-Pick type C disease	Filipin test
Gaucher disease	Leukocyte glucocerebrosidase activity
Alpha-1-antitrypsin deficiency	Detection of PiZ allele

PCR = polymerase chain reaction.
